# Retraction Note: Long non-coding RNA ZNFX1-AS1 promotes the tumor progression and metastasis of colorectal cancer by acting as a competing endogenous RNA of miR-144 to regulate EZH2 expression

**DOI:** 10.1038/s41419-026-09423-4

**Published:** 2026-11-13

**Authors:** Liangliang Shi, Xiaohua Hong, Li Ba, Xiaoxiao He, Yin Xiong, Qian Ding, Shengli Yang, Gang Peng

**Affiliations:** Cancer Center, Union Hospital, Tongji Medical College, Huazhong University of Science and Technology, Wuhan, China

Retraction Note to: *Cell Death and Disease* https://doi.org/10.1038/s41419-019-1332-8, published online 15 February 2019

The Editors-in-Chief have retracted this article.

After publication, concerns were raised regarding the figures presented in this article, specifically:

- Fig. 2c appears highly similar to Figure 7D in [1];
- Fig. 7f appears highly similar to Figure 2E in [2].

Further checks by the publisher have found that:

- Fig. 2c appears highly similar to Fig. 5f;
- Migration ZNFX1-AS1 siRNAs#1 and ZNFX1-AS1 siRNAs#2 in Fig. 3a show high similarity;
- Fig. 3f appears highly similar to Figure 5C in [3].

The authors were unable to provide the raw data. The Editors-in-Chief therefore no longer have confidence in the presented data.

Authors have not responded to correspondence regarding this retraction.
